# The expression and prognostic value of toll-like receptors (TLRs) in pancreatic cancer patients treated with neoadjuvant therapy

**DOI:** 10.1371/journal.pone.0267792

**Published:** 2022-05-10

**Authors:** Anna Maria Nurmi, Jaana Hagström, Harri Mustonen, Hanna Seppänen, Caj Haglund

**Affiliations:** 1 Department of Surgery, Helsinki University Hospital, University of Helsinki, Helsinki, Finland; 2 Department of Pathology, Helsinki University Hospital, University of Helsinki, Helsinki, Finland; 3 Department of Oral Pathology and Radiology, University of Turku, Turku, Finland; 4 Translational Cancer Medicine Research Program, Faculty of Medicine, University of Helsinki, Helsinki, Finland; Texas Tech University Health Science, Lubbock, UNITED STATES

## Abstract

**Objectives:**

Toll-like receptors (TLRs) play a pivotal role in the immune system and carcinogenesis. There is no research on TLR expression and association with survival among preoperatively treated pancreatic cancer patients. We studied the expression intensity and prognostic value of TLRs in pancreatic cancer patients treated with neoadjuvant therapy (NAT) and compared the results to patients undergoing upfront surgery (US).

**Method:**

Between 2000 and 2015, 71 borderline resectable patients were treated with NAT and surgery and 145 resectable patients underwent upfront surgery at Helsinki University Hospital, Finland. We immunostained TLRs 1–5, 7, and 9 on sections of tissue-microarray. We classified TLR expression as 0 (negative), 1 (mild), 2 (moderate), or 3 (strong) and divided into high (2–3) and low (0–1) expression for statistical purposes.

**Results:**

Among TLRs 1, 3, and 9 (TLR1 81% vs 70%, *p* = 0.008; TLR3 92% vs 68%, *p* = 0.001; TLR9 cytoplasmic 83% vs 42%, *p*<0.001; TLR9 membranous 53% vs 25%, *p* = 0.002) NAT patients exhibited a higher immunopositivity score more frequently than patients undergoing upfront surgery. Among NAT patients, a high expression of TLR1 [Hazards ratio (HR) 0.48, p<0.05] associated with a longer postoperative survival, whereas among US patients, high expression of TLR5 (HR 0.64, p<0.05), TLR7 (HR 0.59, p<0.01, and both TLR7 and TLR9 (HR 0.5, p<0.01) predicted a favorable postoperative outcome in separate analysis adjusted for background variables.

**Conclusions:**

We found higher immunopositive intensities among TLRs 1, 3, and 9 in NAT patients. A high TLR1 expression associated with a longer survival among NAT patients, however, among US patients, high expression intensity of TLR5 and TLR7 predicted a favorable postoperative outcome in the adjusted analysis.

## Introduction

Pancreatic ductal adenocarcinoma (PDAC) remains a major challenge and one of the most fatal cancers with a less than 8% 5-year survival rate [1]. Multimodal treatment has emerged as the most effective treatment option, although only 15% to 20% of diagnosed patients are considered resectable [2]. Preoperative oncological therapy, such as neoadjuvant therapy (NAT), can increase the likelihood of radical resections in borderline resectable PDAC patients [3, 4] and downstage locally advanced tumors [5, 6], thus, improving survival [7, 8].

Carcinogenesis and tumor progression induce an inflammatory response both locally and systematically that appears to further promote tumor progression, increase angiogenesis and local immunosuppression [9–11]. The role of inflammation and immune cells in PDAC has been widely examined and the results suggest that inflammation plays a major role in pancreatic carcinogenesis [12, 13]. PDAC is characterized by stromal infiltration containing a variety of inflammatory cells. NAT, however, appears to affect the tumor and its microenvironment and, thus, the cancer-related inflammatory response [14].

Toll-like receptors (TLRs) play a crucial role in the immune system, recognizing molecular patterns and initiating and strengthening local inflammation responses [15]. TLRs have been associated with various cancers, including lung and colon cancer [16, 17]. Ten different TLRs (1 to 10) have been reported in humans [18]. TLRs activate two major intracellular signaling pathways: the MyD88-dependent pathway and the TRIF pathway [19, 20], further activating the NF-kB, MAPK, and interferon regulatory factors [15]. Other TLRs except TLR3 can activate the MyD88-dependent pathway. However, the TRIF pathway can only be activated by TLR3 and TLR4 [20]. Both the MyD88 and TRIF pathways have been associated with pancreatic carcinogenesis [21, 22]. Generally, TLRs are not present in a normal pancreas, although they are expressed in PanINs and PDAC [23, 24] and are related to cancer progression and metastatic potential [25]. TLRs are also involved in several risk factors for PDAC, such as chronic pancreatitis, diabetes, and obesity [26–28]. The role of TLRs in carcinogenesis, however, remains controversial vis-à-vis their pro- and anti-tumor effects [15]. Additionally, cancer can cause a cancer-related systemic inflammatory response and an elevated C-reactive protein (CRP) level has been associated with decreased survival in pancreatic cancer patients [29], however, the association between TLR expression and CRP level has not been established in PDAC patients.

To our knowledge, no studies exist on TLR tumor expression in PDAC patients treated with NAT. Therefore, this study aimed to examine TLRs 1, 2, 3, 4, 5, 7, and 9 among PDAC patients treated with multiple NAT regimens. Additionally, patients undergoing upfront surgery were studied. By studying TLRs in this setting we aimed to investigate the possible immunobiological effects of neoadjuvant therapy, in general, on tumor cells and look at the possible prognostic value TLRs have in PDAC. We hypothesized that due to effects of NAT, the TLR expression would differ between NAT patients and those undergoing upfront surgery.

## Materials and methods

### Patients and data acquisition

**Fig 1:**
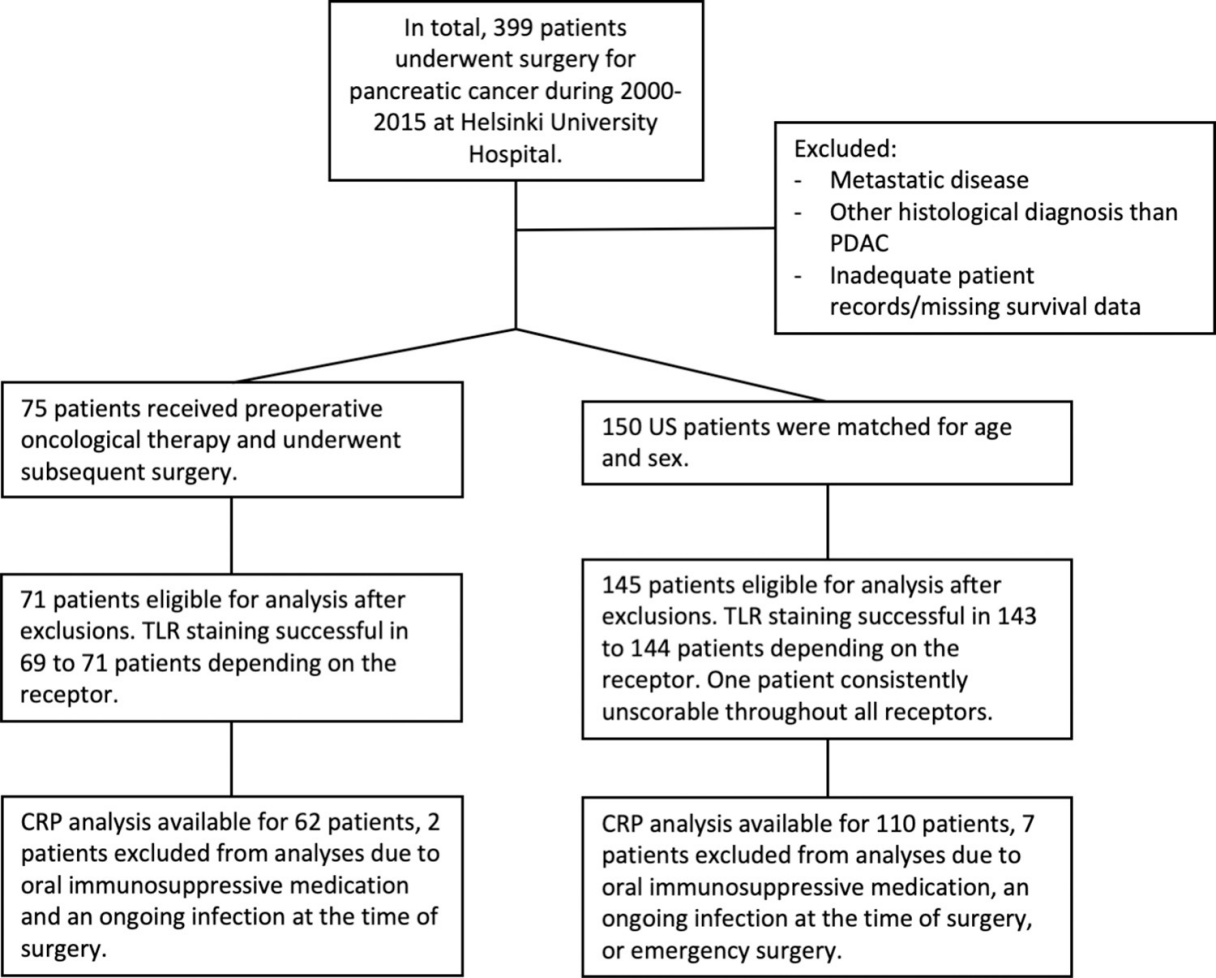
Flow chart of patients. This figure shows the number of patients at each stage of analysis. US = upfront surgery.

A search for PDAC patients in the Helsinki University Hospital database resulted in 399 consecutive patients operated on between July 2000 and December 2015. In total, we included in this study 75 patients treated with NAT and subsequent surgery and 150 resectable patients matched for age and sex who underwent upfront surgery (US). We excluded patients who died from surgery-related complications, and those for whom we had inadequate samples for tissue microarray (total n = 9). The sample size was limited by these constraints in data acquisition. Fig 1 shows the inclusion of patients at each stage. Survival data were collected from patient records and the Finnish Population Registry. Death certificates were obtained from Statistics Finland. This study adhered to the principles of the Declaration of Helsinki. The Surgical Ethics Committee of Helsinki University Hospital (226/E6/2006, extensions 4/17/2013 and 3/27/2019) and the National Supervisory Authority of Health and Welfare approved the study. All patients signed a written informed consent form. The recommendations for reporting tumor markers were applied [30].

### Neoadjuvant therapy (NAT) and adjuvant therapy

Patients treated with NAT had borderline resectable disease upon diagnosis, which was defined as superior mesenteric vein, portal vein, or superior mesenteric artery contact. NAT consisted of FOLFIRINOX, gemcitabine alone or combined with cisplatin, capecitabine or nab-paclitaxel. Additional radiotherapy was administered to 23 (32%) NAT patients. NAT and adjuvant therapy regimens are described in S1 Table. In the NAT group 47 patients (66%) received additional adjuvant therapy and in the US group 100 patients (69%) received adjuvant therapy.

### Tissue microarray (TMA)

Formalin-fixed and paraffin-embedded surgical tissue samples were obtained from the archives of the Department of Pathology, Helsinki University Hospital. The PDAC diagnosis was confirmed by an experienced pathologist (JH) through re-evaluation of all tissue samples. The applied TMA technique has been described in detail elsewhere [31]. Representative areas of the tumor cells were marked on hematoxylin- and eosin-stained slides. Of these tumor cell areas, six 1.0-mm-diameter punches were taken from each tissue sample and mounted on a recipient paraffin block with a semiautomatic TMA instrument (Beecher Instruments, Silver Spring, MD, USA).

### Immunohistochemistry

The TMA blocks were freshly cut into 4-μm-thick sections. After xylene deparaffinization and rehydration through a gradually decreasing concentration of ethanol to distilled water, the slides were treated in a PreTreatment module (Lab Vision Corp, Fremont, CA, USA) in a Tris-HCl pre-treatment buffer (pH 8.5) with TLRs 1, 2 and 4 and in Tris-EDTA (pH 9.0) with TLRs 3, 5, 7, and 9 for 20 min at 98°C for antigen retrieval. For the staining procedure, an Autostainer 480 (Lab Vision) was used. The antigen–antibody reaction was visualized using the Dako REAL EnVision Detection system, Peroxidase/DAB+, Rabbit/Mouse (Dako, Glostrup, Denmark). Details on the antibodies used are described in S2 Table. In every staining series, samples of skin and pharyngeal and palatine tonsils served as positive controls. The stainings were scored independently by AN and JH, who were blinded to both data and outcome. Any differences in scoring were discussed until agreement was reached. Among all studied TLRs, cytoplasmic staining of the tumor cells was scored based on the intensity as negative (0), mild (1), moderate (2), and strong (3). Stroma or other cellular components apart from tumor cells were not scored or analyzed. When present, nuclear staining of tumor cells was scored as negative or positive (TLRs 2, 4, and 5). TLR9 showed distinctively different intensities in membranous staining of tumor cells and was scored as cytoplasmic staining, as negative (0), mild (1), moderate (2), and strong (3). Patients with varying scores among the six TMA punches were classified according to the highest score.

### C-reactive protein (CRP)

The relationship between the TLR expression and circulating CRP was determined based on a high-sensitivity CRP assay that was determined from preoperatively collected plasma samples (n = 172). The high-sensitivity CRP method was described previously [29]. We excluded from CRP analysis patients receiving oral immunosuppressive medication, those with an ongoing infection at the time of surgery or undergoing emergency surgery (total n = 9).

### Statistical analysis

The TLR expression categories were divided into low (0–1) and high (2–3) for statistical analysis. The Fisher’s exact test and linear-by-linear association were used for categorical variables. Continuous variables were examined using the Mann–Whitney U test. The Spearman’s rank correlation test was used for correlations between TLR expressions. Survival was estimated using the Kaplan–Meier method (log rank test). Survival was calculated from the date of surgery. The primary endpoint was death from PDAC (disease-specific survival, DSS), while the secondary endpoint was disease progression or death from PDAC (disease-free survival, DFS). Multivariate analysis was calculated using the Cox proportional hazards method. Based on univariate analyses, age, sex, stage, adjuvant therapy, and perivascular invasion were included in the model as backround variables; every TLR was calculated in a separate adjusted multivariate model. For each variable, the assumption of a constant proportional hazard rate over time was tested using a time-dependent variable; all variables met the assumption. The minimum follow-up period was 4 years or until death. Patients with missing data were excluded from survival analyses. We considered a *p* <0.05 as statistically significant. All tests were two-sided. All statistical analyses were calculated using SPSS version 24.0 (IBM SPSS Statistics, version 24.0; SPSS, Inc. Chicago, IL, USA, an IBM Company).

## Results

After exclusions, 71 NAT patients and 145 patients that underwent upfront surgery were analyzed throughout the study. The clinicopathological characteristics appear in Table 1. Depending upon the receptor, TLR staining was successful in 69 to 71 NAT patients, and in 143 to 144 patients who underwent upfront surgery. Among patients who underwent upfront surgery, we were unable to consistently score one patient across all TLRs. The median follow-up time was 2.3 years.

**Table 1:** Clinicopathological characteristics for NAT and US patients.

	NAT (n = 71)	US (n = 145)	*p*-value
**Age at operation, median (range)**	65 (40–82)	65 (44–82)	0.869
≥65 years	36 (51%)	74 (51%)	0.964
**Gender, female**	40 (56%)	80 (55%)	0.885
**pTN (AJCC 8**^**th**^ **edition)**			
T0	3 (4%)	0	**0.001**
T1	20 (28%)	18 (13%)	
T2	41 (58%)	102 (70%)	
T3	6 (9%)	25 (17%)	
T4	1 (1%)	0	
N0	36 (51%)	40 (28%)	**<0.001**
N1	26 (37%)	65 (44%)	
N2	9 (12%)	40 (28%)	
**Stage (AJCC 8**^**th**^ **edition)**			
0	2 (3%)	0	**<0.001**
IA	10 (14%)	9 (6%)	
IB	22 (31%)	28 (19%)	
IIA	1 (1%)	3 (2%)	
IIB	26 (37%)	65 (45%)	
III	10 (14%)	40 (28%)	
**pTumor size (mm), median (IQR)**	25 (20–30)	30 (25–40)	**0.001**
Grade*			
1	12 (17%)	25 (17%)	0.936
2	44 (62%)	96 (66%)	
3	12 (17%)	24 (17%)	
**R0 resection**	56 (79%)	103 (71%)	0.289
Missing	3 (4%)	8 (6%)	
**Vascular resection**	33 (46%)	49 (34%)	0.076
**Perineural invasion**	45 (63%)	115 (79%)	**0.012**
Missing	0	1 (1%)	
**Perivascular invasion**	15 (21%)	56 (39%)	**0.009**
Missing	0	1 (1%)	
**Postoperative adjuvant therapy**	47 (66%)	100 (69%)	0.301
Discontinuation**	13 (28%)	35 (35%)	
**Cause of death**			
PDAC	54 (76%)	115 (79%)	0.416
Other	2 (3%)	6 (4%)	
Alive	15 (21%)	24 (17%)	
**DSS, months (95% CI)**	30 (25–35)	27 (19–35)	0.658
**DFS, months (95% CI)**	14 (10–19)	12 (9–15)	0.363

Fisher’s exact test and linear-by-linear association (pTN, stage, and tumor grade) for categorical variables, Mann Whitney U test for continuous variables and Kaplan-Meier for survival analysis. Pathological characteristics of NAT patients were gathered from surgical samples, thus, after neoadjuvant therapy. NAT = Neoadjuvant therapy, US = Upfront surgery, AJCC = American Joint Committee on Cancer, DSS = disease-specific survival, DFS = disease-free survival.

*Histological tumor grade missing in 3 patients due to complete response.

**Discontinuation of adjuvant therapy due to adverse effects or disease progression (NAT vs US p = 0.452).

### Expression patterns

**Fig 2:**
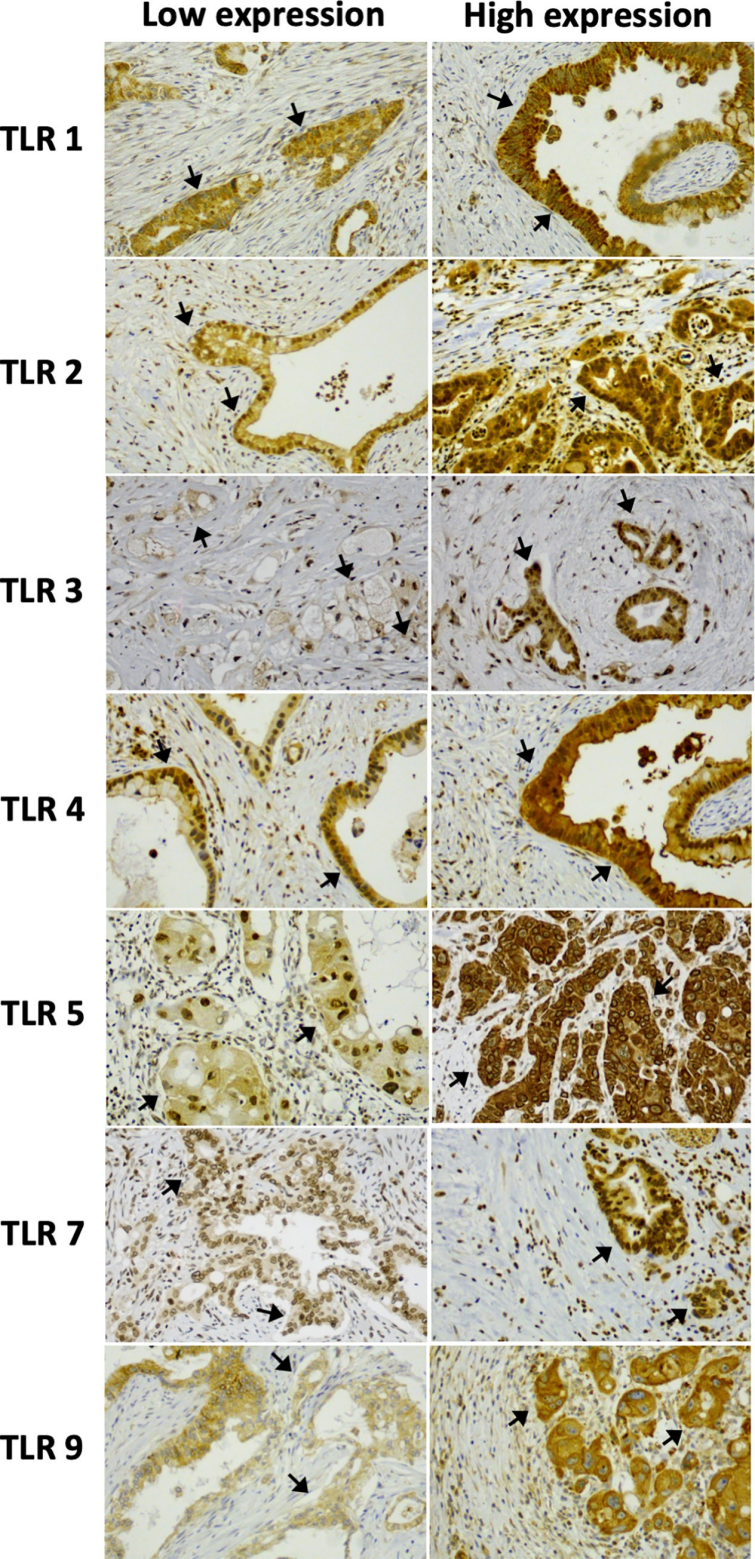
Expression intensity for each TLR separately. Low expression includes intensities 0 (negative) and 1 (mild), high expression intensity includes intensities 2 (moderate) and 3 (strong). Arrows point at tumor cells. Magnification is 20x.

Table 2 shows the distribution of the expression intensity for each TLR and patient group. In addition to the cytoplasmic staining for all TLRs, TLRs 2, 4, and 5 showed nuclear (TLR2: 69% NAT and 84% US; TLR4: 54% NAT and 56% US; TLR5 83% NAT and 77% US) and TLR9 membranous staining (69% NAT and 61% US). For TLR7, cytoplasmic staining and staining on the nuclear membrane were assessed together. TLR5 stained negative in 9% (NAT) to 15% (US) of the patients, while in other receptors negative cytoplasmic expression was rare (0% to 4%; Table 2). Fig 2 shows pictures of low and high expression for every studied TLR.

**Table 2:** TLR staining intensity compared between NAT and US patients for each TLR.

	NAT (n = 71)	US (n = 145)	*p*-value
**TLR1 staining intensity,** 0	0	4 (3%)	
1	13 (18%)	37 (26%)	**0.008**
2	35 (50%)	79 (54%)	
3	22 (31%)	23 (16%)	
**TLR2 staining intensity,** 0	1 (1%)	3 (2%)	
1	12 (17%)	18 (12%)	1.000
2	31 (44%)	72 (50%)	
3	27 (28%)	50 (35%)	
**TLR3 staining intensity,** 0	1 (1%)	1 (1%)	
1	3 (4%)	44 (30%)	**0.001**
2	50 (71%)	78 (54%)	
3	15 (21%)	20 (14%)	
**TLR4 staining intensity,** 0	0	1 (1%)	
1	10 (14%)	21 (14%)	0.371
2	38 (54%)	86 (60%)	
3	22 (31%)	35 (24%)	
**TLR5 staining intensity,** 0	6 (9%)	22 (15%)	
1	29 (41%)	50 (35%)	0.777
2	35 (49%)	63 (43%)	
3	1 (1%)	8 (6%)	
**TLR7 staining intensity,** 0	3 (4%)	1 (1%)	
1	19 (27%)	59 (41%)	0.660
2	44 (62%)	69 (48%)	
3	5 (7%)	14 (9%)	
**TLR9 staining intensity,** 0 **cytoplasm**	0	4 (3%)	
1	11 (16%)	79 (54%)	**<0.001**
2	45 (63%)	46 (32%)	
3	14 (20%)	15 (10%)	
**TLR9 staining intensity,** 0 **membrane**	21 (30%)	56 (39%)	
1	12 (17%)	51 (35%)	**0.002**
2	29 (41%)	31 (21%)	
3	8 (12%)	6 (4%)	

Staining intensity was scored from 0 (negative) to 3 (strong). Linear-by-linear association was used for each TLR to compare expression intensity between NAT and US patients. NAT = Neoadjuvant therapy, US = Upfront surgery. Inadequate samples in NAT patients: 1 in TLRs 1, 4, and 9 and 2 in TLR3; in US patients: 2 in TLRs 1–5, and 1 in TLRs 7 and 9.

TLRs 1, 3, and 9 stained with a high (2–3) intensity more frequently in NAT patients than in patients undergoing upfront surgery (Table 2). The staining intensity for TLRs 2, 4, 5, or 7 did not differ between the patient groups (Table 2).

Among NAT patients, no TLR expression intensity associated with the stage (IA–IIA vs. IIB–III; S3 Table) or NAT regimen (radiation-based vs. chemo-only; S4 Table). However, a high TLR7 expression (2–3) was more frequent among patients undergoing upfront surgery with stage IA–IIA disease than among patients with stage IIB–III disease (S3 Table). Additionally, a correlation analysis was conducted between different cytoplasmic TLR expressions (Table 3). There was a moderately positive (r_s_ = 0.5-<0.7) correlation among NAT patients between TLR2 and TLR5 expression (r_s_ = 0.514, p<0.001). Otherwise correlations were of low positive (r_s_ = 0.3-<0.5) and negligible positive (r_s_<0.3) value.

**Table 3:** Correlation of TLRs 1, 2, 3, 4, 5, 7, and 9 among patients treated with neoadjuvant therapy and upfront surgery.

**NAT**	**TLR1**		**TLR2**		**TLR3**		**TLR4**		**TLR5**		**TLR7**	
	r_s_	p-value	r_s_	p-value	r_s_	p-value	r_s_	p-value	r_s_	p-value	r_s_	p-value
TLR2	0.423	<0.001										
TLR3	0.150	0.219	0.253	0.036								
TLR4	0.287	0.016	0.206	0.086	-0.08	0.514						
TLR5	0.352	0.003	**0.514**	**<0.001**	0.009	0.942	0.280	0.019				
TLR7	0.278	0.020	0.374	0.001	0.082	0.501	0.139	0.252	0.498	<0.001		
TLR9	0.359	0.002	0.431	<0.001	0.298	0.013	0.389	0.001	0.277	0.020	0.361	0.002
**US**	**TLR1**		**TLR2**		**TLR3**		**TLR4**		**TLR5**		**TLR7**	
	r_s_	p-value	r_s_	p-value	r_s_	p-value	r_s_	p-value	r_s_	p-value	r_s_	p-value
TLR2	0.194	0.020										
TLR3	0.333	<0.001	0.329	<0.001								
TLR4	0.161	0.055	0.198	0.018	0.136	0.106						
TLR5	0.350	<0.001	0.294	<0.001	0.415	<0.001	0.104	0.216				
TLR7	0.303	<0.001	0.402	<0.001	0.357	<0.001	0.152	0.070	0.377	<0.001		
TLR9	0.307	<0.001	0.339	<0.001	0.389	<0.001	0.200	0.017	0.356	<0.001	0.319	<0.001

**NAT = neoadjuvant therapy, US = upfront surgery.** TLR = Toll like.

receptor, r_s_ = Spearman’s rank correlation coefficient.

We found no association between TLR expression and preoperative CRP, except for TLR7 expression in patients who underwent upfront surgery (S5 Table). Specifically, the higher the immunointensity was, the lower the CRP value (S5 Table).

### TLR expression and survival

**Fig 3:**
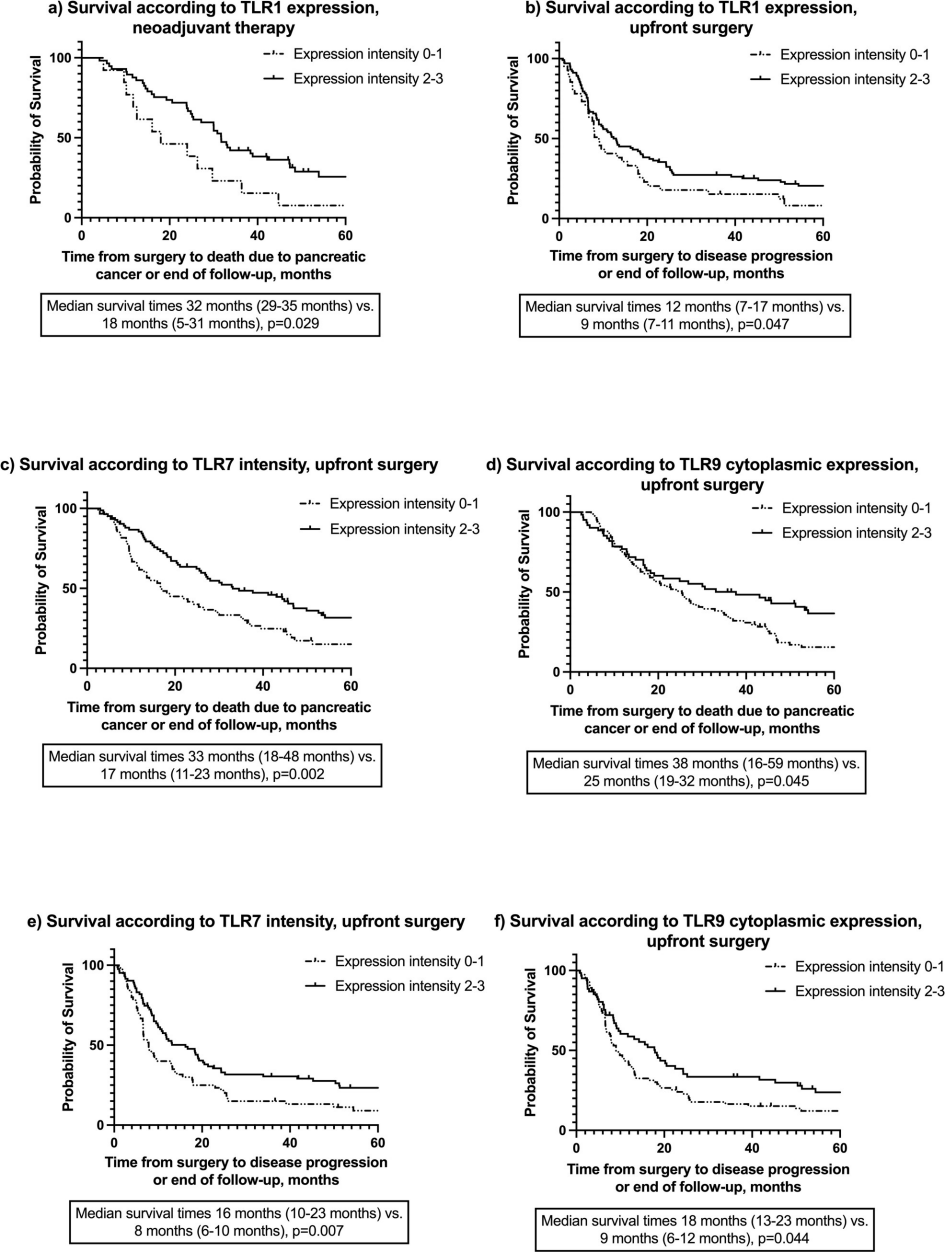
Representation of Kaplan-Meier analysis of TLR1 in NAT patients and TLRs 1, 7, and 9 in upfront resected patients a) Kaplan-Meier analysis of TLR1 expression in NAT patients, disease-specific survival b) Kaplan-Meier analysis of TLR1 expression in upfront resected patients, disease-free survival c) Kaplan-Meier analysis of TLR7 expression in upfront resected patients, disease-specific survival d) Kaplan-Meier analysis of TLR9 cytoplasmic expression in upfront resected patients, disease-specific survival e) Kaplan-Meier analysis of TLR7 expression in upfront resected patients, disease-free survival. f) Kaplan-Meier analysis of TLR9 cytoplasmic expression in upfront resected patients, disease-free survival.

Among NAT patients, a high (2–3) TLR1 expression associated with a longer DSS than low (0–1) expression in the Kaplan-Meier analysis (Fig 3; S6 Table). No other TLR associated with DSS or DFS in the Kaplan–Meier analysis (S6 Table). In patients undergoing upfront surgery, a high (2–3) TLR7 and a high cytoplasmic TLR9 expression presented with a longer DSS and DFS than low (0–1) expression in the Kaplan–Meier analysis along with a high (2–3) TLR1 expression with a longer DFS (Fig 3; S6 Table).

In a grouped analysis, the negativity for one or more receptors in TLRs 1, 3, 7, or 9 in patients undergoing upfront surgery (7 out of 145 patients) predicted a shorter survival [14 months (95% CI 7–21 months) vs 27 months (95% CI 21–34 months), *p =* 0.040]. Among NAT patients, the negative expression of one or more of these receptors did not associate with survival [30 months (95% CI 24–36 months) vs 43 months (95% CI could not be calculated due to low number of patients), *p =* 0.300]. Negativity for at least one of the receptors was detected only in 4 of 71 NAT patients.

### Univariate and multivariate analysis

**Fig 4:**
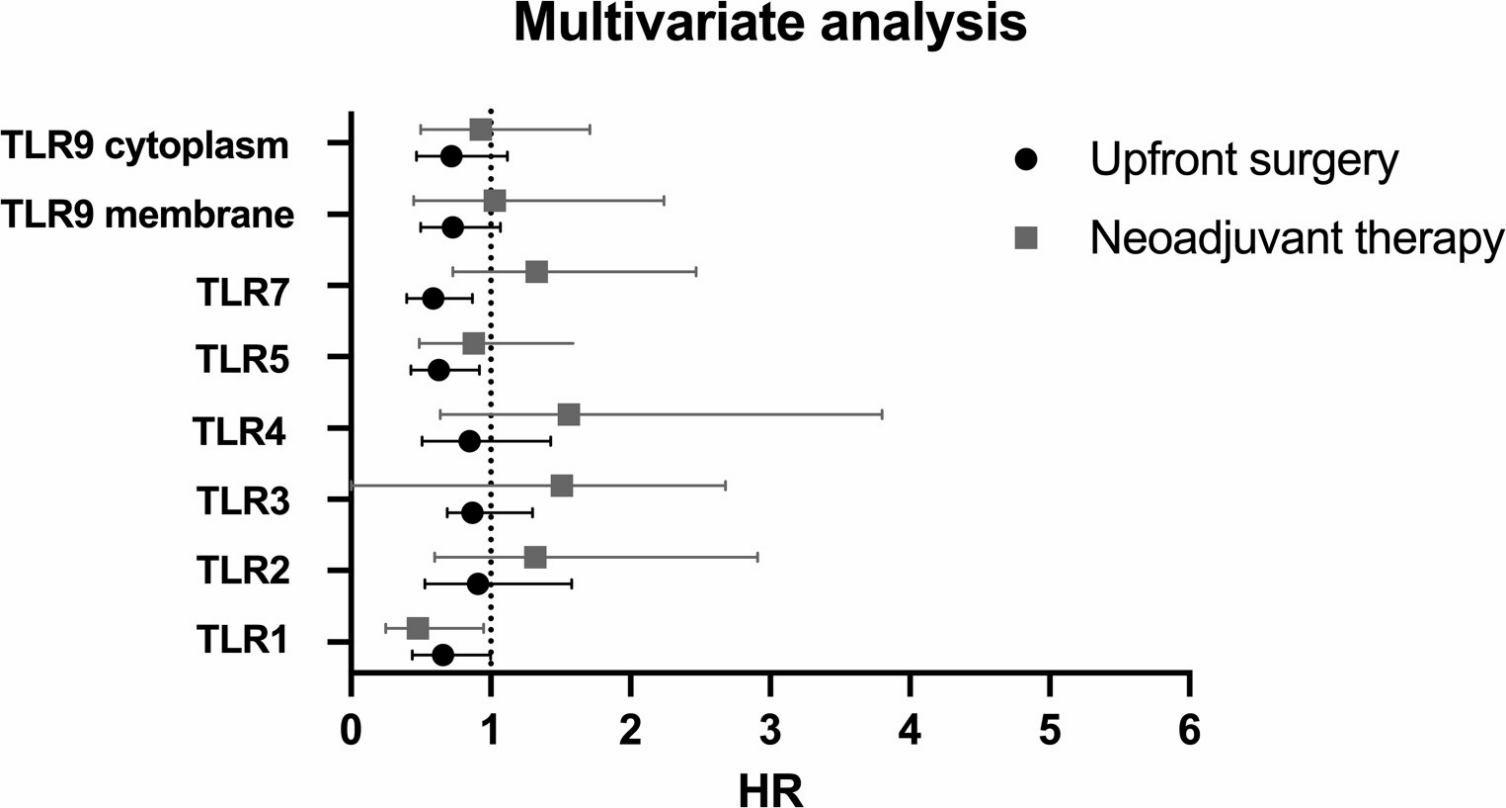
Representation of hazard ratios of the adjusted analysis for each TLR. Every TLR was tested in a separate multivariate analysis for NAT patients and those undergoing upfront surgery separately. Multivariate analysis included age, sex, stage (IA-IIA vs IIB-III), perivascular invasion, adjuvant therapy and TLR expression intensity (high expression vs low expression). X-axis shows calculated hazard ratio with 95% CI. End-point was disease-specific death. HR = Hazards ratio.

Among NAT patients, adjuvant therapy and high TLR1 expression were markers of a favorable prognosis in univariate analysis (S7 Table) and the high expression of TLR1 along with adjuvant therapy predicted a favorable outcome in the adjusted analysis (Fig 4; S8 Table).

Among patients undergoing upfront surgery, stage, perivascular invasion, tumor differentiation grade 3, a high expression of TLR7, and a high expression of cytoplasmic TLR9 emerged as prognostic markers in univariate analysis (S7 Table). In the adjusted model tested for each TLR separately, TLR5 and TLR7 emerged as independent prognostic factors along with stage, adjuvant therapy and perivascular invasion (Fig 4; S8 Table). An additional adjusted multivariate model was conducted with a combination variable of TLR7 and TLR9 for their significance in univariate analysis. There was a significant correlation between TLR7 and TLR9 (r_s_ = 0.32, p<0.001), therefore, a combination variable was employed (both TLR7 and TLR9 high, either TLR7 or TLR9 high, both TLR7 and TLR9 low). Compared to low expression in both, when both TLR7 and TLR9 were high, the hazard ratio was at 0.50 (95% CI 0.30–0.82, p = 0.006) (S9 Table).

## Discussion

PDAC stands as one of the most complex cancers to tackle and survival seems to depend on both patient- and tumor-related factors. PDAC is distinctive given its tumor-promoting microenvironment with hypoxic conditions and chronic local inflammation, factors stimulating TLR-activated pathways. To the best of our knowledge, this study represents the first to determine the expression of TLRs in PDAC patients treated with NAT. With this study, we wanted to search the possible immunobiological effects of neoadjuvant therapy on the tumor cells by observing TLR expression and comparing that to treatment-naïve tumors. Altogether, there is little evidence on the biology of PDAC in patients treated with NAT. Here, we show that TLRs 1, 3, and 9 all expressed at a high intensity more frequently among patients treated with NAT than among those undergoing upfront surgery. Only TLR1 expression associated with survival among NAT patients. This might be due to a number of reasons, a small patient number, differences in disease stage, the effects of NAT on TLR signaling or on survival within the NAT group. However, it is of interesting novel value since this has not been studied before. Among patients undergoing upfront surgery, the high expression of TLRs 7 and 9 associated with a better survival than the low expression in the Kaplan-Meier analysis and TLR5 and TLR7 in adjusted Cox analysis.

As we hypothesized, the TLR expression intensities of tumor cells differed in NAT patients and those undergoing upfront surgery. NAT causes a local inflammatory reaction affecting the tumor, its microenvironment and, thus, the cancer-related inflammatory response [14]. TLRs are activated by both endogenous damage-associated molecular patterns (DAMPs) and exogenous pathogen-associated molecular patterns (PAMPs). Based on the effects of NAT, it most likely increases the DAMP-mediated activation. These factors could contribute to the fact that the expression intensities of TLRs varied between NAT patients and those who underwent upfront surgery, a finding previously not reported. The PDAC microenvironment includes various inflammatory components, such as tumor-associated macrophages, mast cells, and T cells in addition to pancreatic stellate cells that form the stroma and create the hypovasculature [13, 32, 33]. PDAC cells need to survive under an extremely hypoxic environment, with different vascularization on the periphery of the tumor versus in the areas with a dense desmoplastic reaction [34, 35]. Hypoxic conditions activate different genetic and metabolic changes, thereby helping the cancer cells to survive [34, 35]. By theory, these conditions could cause endogenous DAMPs to disperse, causing the activation of TLR-mediated signaling.

Along with effects on tumor cells, NAT appears to affect the immune cell infiltrate in the tumor [14]. Additionally, TLR activation has been linked to stromal inflammation and fibrosis [21]. This supports our finding that the expression of TLRs 1, 3, and 9 were higher among NAT patients, since NAT causes fibrosis in the pancreas. This, in turn, causes changes in the tumor microenvironment and, thus, TLR expression subsequently differs between NAT patients and those undergoing upfront surgery. However, distinguishing between treatment-related fibrosis and reactive fibrosis due to the cancer remains difficult [36].

TLRs 1, 2, 4, and 5 are normally expressed on the cell membranes while TLRs 3, 7, and 9 are normally expressed on cell organelles, such as endosomes [37]. Furthermore, TLRs appear to exist on both primary and metastatic tumor sites in PDAC [38]. The alternating expression cites in cancer cells have been postulated to result from the effects of TLRs on carcinogenesis [39]. In this study, the expression patterns in the tumor differed from those under normal, physiological conditions. TLR9 was visible on the cell membrane along with the cytoplasmic expression, and TLR1 was expressed solely in the cytoplasm. Along with cytoplasmic staining TLR7 was expressed on the nuclear membrane with visible differences from negative to strong intensity. TLRs 2, 4, and 5 also showed nuclear expression, suggesting a possible oncogenic impact. This agrees with previous findings, indicating that TLR-mediated signaling in cancer differs from normal conditions [40].

Because of their expression at both primary and metastatic sites and their studied implications, TLRs 2, 4, 7, and 9 have been studied as potential targets for adjuvant therapy [38, 41]. Grimming et al. showed that TLR7 and TLR8 are partially responsible for chemoresistance in human PDAC models, suggesting that the targeting of TLR signaling may possibly reduce chemoresistance [42]. Additionally, TLR7 expression has been linked to tumor progression, inflammation, and decreasing the anti-tumoral molecules in murine models and overexpression of TLR7 has been reported in human PDAC [41]. However, TLR7 expression appears to increase the number of cytotoxic immune cells in both murine and human pancreatic cancer specimen [41, 43, 44]. Interestingly, our results showed that a high TLR7 expression among patients who underwent upfront surgery associated with a longer survival. Patients with a high expression, however, were more likely to have early stage disease. This might affect survival data. Additionally, the higher the TLR7 expression intensity was, the lower the CRP level was. These factors could be linked, although we could not establish that connection here. A high CRP was previously found to shorten survival in PDAC [31]. Thus, we can speculate that being cytoplasmic and on the nuclear membrane (not on the cell membrane), TLR7 acts differently and does not induce responses similar to inflammation caused by PAMPs.

Zambrinis et al. demonstrated in a murine model of pancreatic stellate cells that TLR9 is expressed during PDAC tumorigenesis and carries immunosuppressive effects in the tumor microenvironment [45]. However, our study showed that a high TLR9 expression in patients who underwent upfront surgery associated with a longer survival than a low expression. In addition to methodological variation, this could indicate that the effects of TLR signaling on tumor progression and survival depend upon the stage, being different in early carcinogenesis and fully formed cancer. Furthermore, based on a small patient series and cell line work, Grimming et al. concluded that TLR expression and signaling were closely associated with inflammation-mediated cancer cell proliferation, tumor progression, and, thus, the metastatic potential [46]. This inconclusive data on different TLRs could result from their shifting effects during different stages of PDAC and precursors, tumorigenesis, and tumor progression as well as patient-related factors. These contradictory results also suggest that TLRs behave differently *in vivo*, and *in vitro* setups are insufficient to explain the precise relationships. Additionally, TLRs seem to act synergistically generating different cytokine responses with different TLR combinations [47].

In lung and colon cancer [16, 17], a high TLR expression associates with a poor prognosis, although results in PDAC vary. Similar to our findings, Leppänen et al. concluded, based on an immunohistochemical series of 65 patients, that a high TLR9 expression was associated with a longer patient survival than low expression, whereas TLR2 and TLR4 did not associate with survival [48]. The expression of these TLRs was not associated with the stage, tumor size, lymph node metastases, or tumor necrosis. Although that study separately evaluated membranous, nuclear, and cytoplasmic staining, the results were similar to ours. Interestingly, Zhang et al. discovered that TLR4 is overexpressed in PDAC and based on tumor cell analysis the over-expression correlates with tumor size, lymph node metastases and decreased survival [49]. However, Lanki et al. found a high TLR2 intensity in patients who underwent surgery for PDAC with small tumors (<30 mm) and TLR4 in stage I–II disease served as markers of a favorable prognosis [50]. Even though they used a similar immunohistochemical method as we did, we could not reproduce those findings here. Furthermore, Lanki et al. reported that strong TLR1 expression predicted a longer survival, while the negative expression of TLRs 1, 3, 7, or 9 predicted a shorter survival in PDAC patients who underwent surgery [51]. In our study, a high TLR1 expression associated with a longer survival among NAT patients, however, lacked statistical significance on disease-free survival among upfront resected (p = 0.05). The prognostic value of negativity for TLRs 1, 3, 7, or 9 in patients undergoing upfront surgery was similar in our study. Among NAT patients, the negative expression of one or more of these receptors did not associate with survival. However, negativity for at least one of the receptors was detected only in 4 NAT patients, likely affecting the result. The varying results for different TLR studies could, of course, result from methodological differences, as well as from patient- and tumor-related factors. Additionally, these studies have different inclusion years, a different follow-up period and in our study the upfront surgery patients used as controls were matched for age and sex with the NAT patients.

In addition to the multiple TLRs studied, this study’s strengths include a rather large cohort of PDAC patients with reliable clinicopathological and follow-up data. The advantage of this study also lies in our ability to evaluate patients treated with NAT and to compare them to those undergoing upfront surgery. However, the NAT group is quite small and the treatment regimens are too heterogenous for subpopulation analyses, and, thus, this represents a limitation in our study. Additionally, the stage distribution and percentage of perineural and perivascular invasion differ between studied patient groups, most likely causing bias and complicating the conclusions on the actual effects of neoadjuvant therapy on TLR expression. Furthermore, all patients underwent pancreatic surgery, representing only a subset of PDAC patients. Immunohistochemistry is subjective by nature and this represents another limitation in our study; the use of a more quantitative method warrants for further studies.

PDAC represents a rather complex cancer, thus, complicating the search for well-performing and trustworthy biomarkers. In our study, we demonstrated that among patients treated with NAT, TLRs 1, 3, and 9 more frequently stained with a high expression intensity than the intensity among corresponding patients who underwent upfront surgery. Among NAT patients, only high TLR1 expression associated with survival, whereas, among patients undergoing upfront surgery, a high TLR7 and TLR9 expression both associated with a longer survival. We believe the clinical relevance of TLRs in pancreatic cancer should be investigated more thoroughly in future studies.

## Supporting information

S1 Table Administered neoadjuvant and adjuvant therapy regimens. All regimens, both neoadjuvant and adjuvant therapy, with the number of cycles used for both patients treated with neoadjuvant therapy and upfront surgery. NAT = Neoadjuvant therapy, US = Upfront surgery.
(DOCX)

S2 Table Details on the antibodies used in the study. Antibodies for TLR5 and TLR9 were monoclonal mouse antibodies and for TLRs 1, 2, 3, 4, and 7 polyclonal rabbit antibodies. Parentheses show the dilution ratio.
(DOCX)

S3 Table TLR staining intensity matched against disease stage in NAT and US patients separately. AJCC 8^th^ edition was used for stage. Linear-by-linear association was used. NAT = Neoadjuvant therapy, US = Upfront surgery. *p<0.05.
(DOCX)

S4 Table TLR expression intensity analyzed against neoadjuvant therapy regimen. Neoadjuvant therapy regimens were divided into chemo-only and radiotherapy-based.
(DOCX)

S5 Table Preoperative CRP matched against TLR expression intensity for NAT patients and US patients separately. Jonckheere-Terpstra test was used to determine the difference between median values. Preoperative CRP was available in 60 NAT patients and 103 US patients. NAT = Neoadjuvant therapy, US = Upfront surgery. *Due to low patient number in this patient group, 95% CI’s could not be calculated.
(DOCX)

S6 Table Disease-specific and disease-free survival in months according to TLR staining intensity for NAT and US patients separately. Survival was calculated from surgery to disease progression or death due to pancreatic cancer. Survival was estimated with the Kaplan-Meier method (Log rank). NAT = Neoadjuvant therapy, US = Upfront surgery. *Due to low patient number with low TLR3 staining intensity, the 95% CI’s could not be calculated.
(DOCX)

S7 Table S5 Table. Univariate analysis for US and NAT patients separately. Univariate analysis calculated with the Cox proportional hazards method. End-point was disease-specific death. US = Upfront surgery, NAT = Neoadjuvant therapy. *Male vs female, **Stage IIB III vs IA IIA, ***TLR expression intensity high vs low.
(DOCX)

S8 Table Adjusted analysis for each TLR separately. NAT and US patients were analyzed separately. Multivariate model including TLR expression intensity (for each TLR separately), age, sex, stage, adjuvant therapy and perivascular invasion. ^1^p<0.001, ^2^p<0.01, ^3^p<0.05. *Sex male vs female, **Stage IIB-III vs IA-IIA, ***TLR high expression intensity vs low expression intensity.
(DOCX)

S9 Table Adjusted analysis with combined TLR7 and TLR9 expression. NAT and US patients analyzed separately.
(DOCX)

S1 File Data file. (SAV)
