# Supplementary material for: The expression and prognostic value of toll-like receptors (TLRs) in pancreatic cancer patients treated with neoadjuvant therapy
Source: PLoS One. 2022 May 10;17(5):e0267792. doi: 10.1371/journal.pone.0267792 (PMC9089880; doi:10.1371/journal.pone.0267792)
Supplement: S1 Table — All regimens, both neoadjuvant and adjuvant therapy, with the number of cycles used for both patients treated with neoadjuvant therapy and upfront surgery. NAT = Neoadjuvant therapy, US = Upfront surgery. (DOCX) [file pone.0267792.s001.docx]

**S1 Table. Administered neoadjuvant and adjuvant therapy regimens.**

| **NAT patients – Neoadjuvant therapy regimens (n=71)** | **US patients – Adjuvant therapy regimens (n=100)** |
| --- | --- |
| 10 x Gemcitabine 3-8 cycles | 88 x Gemcitabine 1-6 cycles |
| 23 x Gemcitabine + cisplatin 3-7 cycles | 3 x Gemcitabine chemoradiotherapy |
| 10 x Gemcitabine chemoradiotherapy 50,4 Gy | 2 x Gemcitabine 1 cycle, changed to capecitabine 2-7 cycles |
| 5 x Gemcitabine + cisplatin 3-5 cycles, and gemcitabine chemoradiotherapy 45-60Gy | 1 x Gemcitabine 4 cycles, changed to Folfirinox 2 cycles |
| 3 x Gemcitabine + cisplatin 6-7 cycles, and capecitabine chemoradiotherapy 50,4Gy | 1 x Gemcitabine 3 cycles, changed to capecitabine 8 cycles |
| 3 x Gemcitabine 3-4 cycles, and gemcitabine  chemoradiotherapy 50,4 Gy | 1 x Gemcitabine + nab-paclitaxel 6 cycles |
| 1 x Gemcitabine 1 cycle, gemcitabine + cisplatin 7 cycles, gemcitabine 2 cycles | 4 x Capecitabine 3-7 cycles |
| 1 x Gemcitabine + cisplatin + capecitabine 3 cycles |  |
| 1 x Gemcitabine + capecitabine 3 cycles |  |
| 2 x Gemcitabine + nab-paclitaxel 3-4 cycles |  |
| 7 x Folfirinox 5-8 cycles |  |
| 1 x Folfirinox 2 cycles, gemcitabine + cisplatin 3 cycles, gemcitabine chamoradiotherapy 50,4 Gy |  |
| 1 x Gemcitabine 1 cycle, Nab-paclitaxel + gemcitabine 2 cycles |  |
| 1 x Radiotherapy 50,4 Gy (no chemotherapy) |  |
| 2 x not specified |  |
| **NAT patients – Adjuvant therapy regimens (n=47)** |  |
| 33 x Gemcitabine 1-7 cycles |  |
| 6 x Gemcitabine + cisplatin 3-4 cycles |  |
| 2 x Gemcitabine + cisplatin 1 cycle, reduced to gemcitabine 2-3 cycles |  |
| 1 x Gemcitabine + cisplatin 3 cycle, reduced to gemcitabine 1 cycle |  |
| 1 x Gemcitabine 1 cycle, changed to capecitabine 3 cycles |  |
| 1 x Gemcitabine + capecitabine 2 cycles |  |
| 1 x Capecitabine 8 cycles |  |
| 1 x Folfirinox 7 cycles |  |
| 1 x Folfirinox 1 cycle, changed to gemcitabine 3 cycles |  |

Total number of NAT patients before exclusions was 75, after exclusions 71. Out of these 71 patients, 47 received additional adjuvant therapy. Out of the 150 US patients, 5 were excluded. Out of the 145 US patients analyzed, 100 patients received adjuvant therapy.
