# Supplementary material for: The expression and prognostic value of toll-like receptors (TLRs) in pancreatic cancer patients treated with neoadjuvant therapy
Source: PLoS One. 2022 May 10;17(5):e0267792. doi: 10.1371/journal.pone.0267792 (PMC9089880; doi:10.1371/journal.pone.0267792)
Supplement: S2 Table — Antibodies for TLR5 and TLR9 were monoclonal mouse antibodies and for TLRs 1, 2, 3, 4, and 7 polyclonal rabbit antibodies. Parentheses show the dilution ratio. (DOCX) [file pone.0267792.s002.docx]

**S2 Table. Details on the antibodies used in the study.**

|  | **Antibody** |
| --- | --- |
| **TLR1*** | (H-90): sc-30000 (1:100) |
| **TLR2*** | (H-175): sc-10739 (1:100) |
| **TLR3*** | OABF00700 (1:1500) |
| **TLR4**** | (H-80): sc-10741 (1:50) |
| **TLR5***** | NBP2-24787 (1:100) |
| **TLR7***** | NBP2-24906 (1:300) |
| **TLR9*** | (26C593): sc-52966 (1:300) |

Antibodies for TLR5 and TLR9 were monoclonal mouse antibodies and for TLRs 1, 2, 3, 4, and 7 polyclonal rabbit antibodies. Parentheses show the dilution ratio.

*Santa Cruz Biotechnology, Santa Cruz, CA, USA.

**Aviva Systems Biology, San Diego, CA, USA.

***Novus Biologicals, Littleton, CO, USA.
