# Supplementary material for: The expression and prognostic value of toll-like receptors (TLRs) in pancreatic cancer patients treated with neoadjuvant therapy
Source: PLoS One. 2022 May 10;17(5):e0267792. doi: 10.1371/journal.pone.0267792 (PMC9089880; doi:10.1371/journal.pone.0267792)
Supplement: S3 Table — AJCC 8th edition was used for stage. Linear-by-linear association was used. NAT = Neoadjuvant therapy, US = Upfront surgery. *p<0.05. (DOCX) [file pone.0267792.s003.docx]

**S3 Table. TLR staining intensity matched against disease stage in NAT and US patients separately.**

|  | **NAT (n=71)** | **Stage IA-IIA (n=35)** | **Stage IIB-III (n=36)** | **US (n=145)** | **Stage IA-IIA (n=41)** | **Stage IIB-III (n=104)** |
| --- | --- | --- | --- | --- | --- | --- |
| **TLR 1 staining intensity** |  |  |  |  |  |  |
| 0 | 0 | 0 | 0 | 4 (3%) | 1 (2%) | 3 (3%) |
| 1 | 13 (18%) | 7 (20%) | 6 (17%) | 37 (26%) | 10 (25%) | 27 (26%) |
| 2 | 35 (50%) | 15 (43%) | 20 (55%) | 79 (54%) | 22 (54%) | 57 (55%) |
| 3 | 22 (31%) | 12 (34%) | 10 (28%) | 23 (16%) | 7 (17%) | 16 (15%) |
| Inadequate sample | 1 (1%) | 1 (3%) | 0 | 2 (1%) | 1 (2%) | 1 (1%) |
| **TLR 2 staining intensity, cytoplasm** |  |  |  |  |  |  |
| 0 | 1 (1%) | 0 | 1 (3%) | 3 (2%) | 1 (2%) | 2 (2%) |
| 1 | 12 (17%) | 7 (20%) | 5 (14%) | 18 (12%) | 4 (10%) | 14 (13%) |
| 2 | 31 (44%) | 15 (43%) | 16 (44%) | 72 (50%) | 20 (49%) | 52 (50%) |
| 3 | 27 (28%) | 13 (37%) | 14 (39%) | 50 (35%) | 15 (37%) | 35 (34%) |
| Inadequate sample | 0 | 0 | 0 | 2 (1%) | 1 (2%) | 1 (1%) |
| **TLR 3 staining intensity** |  |  |  |  |  |  |
| 0 | 1 (1%) | 0 | 1 (2%) | 1 (1%) | 0 | 1 (1%) |
| 1 | 3 (4%) | 3 (9%) | 0 | 44 (30%) | 10 (25%) | 34 (33%) |
| 2 | 50 (71%) | 22 (63%) | 28 (78%) | 78 (54%) | 21 (51%) | 57 (55%) |
| 3 | 15 (21%) | 9 (26%) | 6 (18%) | 20 (14%) | 9 (22%) | 11 (10%) |
| Inadequate sample | 2 (3%) | 1 (2%) | 1 (2%) | 2 (1%) | 1 (2%) | 1 (1%) |
| **TLR 4 staining intensity, cytoplasm** |  |  |  |  |  |  |
| 0 | 0 | 0 | 0 | 1 (1%) | 0 | 1 (1%) |
| 1 | 10 (14%) | 7 (20%) | 3 (8%) | 21 (14%) | 6 (15%) | 15 (14%) |
| 2 | 38 (54%) | 14 (40%) | 24 (67%) | 86 (60%) | 28 (68%) | 58 (56%) |
| 3 | 22 (31%) | 13 (37%) | 9 (25%) | 35 (24%) | 6 (15%) | 29 (28%) |
| Inadequate sample | 1 (1%) | 1 (3%) | 0 | 2 (1%) | 1 (2%) | 1 (1%) |
| **TLR 5 staining intensity** |  |  |  |  |  |  |
| 0 | 6 (9%) | 3 (9%) | 3 (8%) | 22 (15%) | 4 (10%) | 18 (17%) |
| 1 | 29 (41%) | 14 (40%) | 15 (42%) | 50 (35%) | 17 (42%) | 33 (32%) |
| 2 | 35 (49%) | 17 (48%) | 18 (50%) | 63 (43%) | 17 (42%) | 46 (44%) |
| 3 | 1 (1%) | 1 (3%) | 0 | 8 (6%) | 2 (4%) | 6 (6%) |
| Inadequate sample | 0 | 0 | 0 | 2 (1%) | 1 (2%) | 1 (1%) |
| **TLR 7 staining intensity** |  |  |  |  |  |  |
| 0 | 3 (4%) | 2 (6%) | 1 (3%) | 1 (1%) | **0** | **1 (1%)*** |
| 1 | 19 (27%) | 9 (26%) | 10 (28%) | 59 (41%) | **8 (20%)** | **51 (49%)** |
| 2 | 44 (62%) | 22 (62%) | 22 (61%) | 69 (48%) | **25 (61%)** | **44 (43%)** |
| 3 | 5 (7%) | 2 (6%) | 3 (8%) | 14 (9%) | **7 (17%)** | **7 (7%)** |
| Inadequate sample | 0 | 0 | 0 | 1 (1%) | **1 (2%)** | **0** |
| **TLR 9 staining intensity, cytoplasm** |  |  |  |  |  |  |
| 0 | 0 | 0 | 0 | 4 (3%) | 0 | 4 (4%) |
| 1 | 11 (16%) | 7 (20%) | 4 (11%) | 79 (54%) | 22 (54%) | 57 (54%) |
| 2 | 45 (63%) | 21 (60%) | 24 (67%) | 46 (32%) | 11 (27%) | 35 (34%) |
| 3 | 14 (20%) | 6 (17%) | 8 (22%) | 15 (10%) | 7 (17%) | 8 (8%) |
| Inadequate sample | 1 (1%) | 1 (3%) | 0 | 1 (1%) | 1 (2%) | 0 |
| **TLR 9 staining intensity, membrane** |  |  |  |  |  |  |
| 0 | 21 (30%) | 12 (34%) | 9 (25%) | 56 (39%) | 18 (44%) | 38 (37%) |
| 1 | 12 (17%) | 7 (20%) | 5 (14%) | 21 (15%) | 11 (27%) | 40 (38%) |
| 2 | 29 (41%) | 10 (29%) | 19 (53%) | 31 (21%) | 7 (17%) | 24 (23%) |
| 3 | 8 (12%) | 5 (14%) | 3 (8%) | 6 (4%) | 4 (10%) | 2 (2%) |
| Inadequate sample | 1 (1%) | 1 (3%) | 0 | 1 (1%) | 1 (2%) | 0 |

AJCC 8^th^ edition was used for stage. Linear-by-linear association was used. NAT=Neoadjuvant therapy, US=Upfront surgery. *p<0.05.
