# Supplementary material for: The expression and prognostic value of toll-like receptors (TLRs) in pancreatic cancer patients treated with neoadjuvant therapy
Source: PLoS One. 2022 May 10;17(5):e0267792. doi: 10.1371/journal.pone.0267792 (PMC9089880; doi:10.1371/journal.pone.0267792)
Supplement: S4 Table — Neoadjuvant therapy regimens were divided into chemo-only and radiotherapy-based. (DOCX) [file pone.0267792.s004.docx]

**S4 Table. TLR expression intensity analyzed against neoadjuvant therapy regimen.**

|  | **Chemo-only** | **Radiotherapy** | **p-value** |
| --- | --- | --- | --- |
| **TLR1** |  |  |  |
| 0-1 | 7 (16%) | 5 (22%) | 0.522 |
| 2-3 | 38 (84%) | 18 (78%) |  |
| **TLR2** |  |  |  |
| 0-1 | 9 (20%) | 4 (17%) | 1.000 |
| 2-3 | 36 (80%) | 20 (83%) |  |
| **TLR3** |  |  |  |
| 0-1 | 3 (7%) | 1 (4%) | 1.000 |
| 2-3 | 41 (93%) | 22 (96%) |  |
| **TLR4** |  |  |  |
| 0-1 | 5 (11%) | 5 (22%) | 0.288 |
| 2-3 | 40 (89%) | 18 (78%) |  |
| **TLR5** |  |  |  |
| **0-1** | **17 (38%)** | **16 (67%)** | **0.026** |
| **2-3** | **28 (62%)** | **8 (33%)** |  |
| **TLR7** |  |  |  |
| 0-1 | 12 (27%) | 9 (38%) | 0.415 |
| 2-3 | 33 (73%) | 15 (62%) |  |
| **TLR9 cytoplasm** |  |  |  |
| 0-1 | 5 (11%) | 6 (26%) | 0.164 |
| 2-3 | 40 (89%) | 17 (74%) |  |
| **TLR9 membranous** |  |  |  |
| 0-1 | 18 (40%) | 14 (61%) | 0.128 |
| 2-3 | 27 (60%) | 9 |  |
