# Supplementary material for: The expression and prognostic value of toll-like receptors (TLRs) in pancreatic cancer patients treated with neoadjuvant therapy
Source: PLoS One. 2022 May 10;17(5):e0267792. doi: 10.1371/journal.pone.0267792 (PMC9089880; doi:10.1371/journal.pone.0267792)
Supplement: S5 Table — Jonckheere-Terpstra test was used to determine the difference between median values. Preoperative CRP was available in 60 NAT patients and 103 US patients. NAT = Neoadjuvant therapy, US = Upfront surgery. *Due to low patient number in this patient group, 95% CI’s could not be calculated. (DOCX) [file pone.0267792.s005.docx]

**S5 Table. Preoperative CRP matched against TLR expression intensity.**

|  | **n** | **NAT (n=60)**  **median, mg/l (IQR)** | ***p-*value** | **n** | **US (n=103)**  **median, mg/l (IQR)** | ***p-*value** |
| --- | --- | --- | --- | --- | --- | --- |
| **TLR1 staining intensity** |  |  |  |  |  |  |
| 0 | 0 | - | 0.404 | 3 | 11.3 (-)* | 0.441 |
| 1 | 11 | 8.9 (1.9-19.8) |  | 28 | 4.1 (1.5-16.2) |  |
| 2 | 28 | 2.4 (1.4-5.9) |  | 52 | 2.9 (1.5-7.6) |  |
| 3 | 20 | 4.8 (1.2-7.4) |  | 19 | 2.6 (1.4-7.9) |  |
| Inadequate sample | 1 |  |  | 1 |  |  |
| **TLR2 staining intensity** |  |  |  |  |  |  |
| 0 | 1 | 1.5 (-)* | 0.356 | 3 | 4.8 (-)* | 0.416 |
| 1 | 11 | 2.3 (1.1-4.5) |  | 10 | 7.8 (1.4-15.9) |  |
| 2 | 26 | 3.6 (1.5-12.1) |  | 56 | 2.9 (1.5-8.3) |  |
| 3 | 22 | 4.8 (1.5-8.4) |  | 33 | 4.1 (1.2-7.6) |  |
| Inadequate sample | 0 |  |  | 1 |  |  |
| **TLR3 staining intensity** |  |  |  |  |  |  |
| 0 | 1 | 1.1 (-)* | 0.418 | 1 | 11.3 (-)* | 0.773 |
| 1 | 3 | 4.4 (-)* |  | 32 | 3.5 (1.5-14.5) |  |
| 2 | 42 | 3.2 (1.6-6.7) |  | 56 | 3.4 (1.3-7.8) |  |
| 3 | 12 | 6.8 (1.1-10.7) |  | 13 | 3.2 (1.6-9.1) |  |
| Inadequate sample | 1 |  |  | 1 |  |  |
| **TLR4 staining intensity** |  |  |  |  |  |  |
| 0 | 0 | - | 0.106 | 1 | 11.3 (-)* | 0.126 |
| 1 | 8 | 4.3 (1.7-15.3) |  | 16 | 4.8 (1.7-14.7) |  |
| 2 | 31 | 4.5 (1.5-9.3) |  | 57 | 3.3 (1.4-12.1) |  |
| 3 | 20 | 1.9 (1.0-5.7) |  | 28 | 2.4 (1.3-5.6) |  |
| Inadequate sample | 1 |  |  | 1 |  |  |
| **TLR5 staining intensity** |  |  |  |  |  |  |
| 0 | 5 | 1.6 (1.3-7.2) | 0.298 | 18 | 2.6 (1.7-13.8) | 0.342 |
| 1 | 23 | 2.3 (0.9-8.0) |  | 37 | 3.6 (1.5-10.4) |  |
| 2 | 31 | 4.7 (1.6-9.3) |  | 44 | 3.3 (1.3-7.2) |  |
| 3 | 1 | 4.4 (-)* |  | 3 | 8.9 (-)* |  |
| Inadequate sample | 0 |  |  | 1 |  |  |
| **TLR7 staining intensity** |  |  |  |  |  |  |
| 0 | 3 | 1.6 (-)* | 0.442 | 1 | 11.3 (-)* | **0.046** |
| 1 | 16 | 1.9 (0.9-9.4) |  | 42 | 4.8 (1.8-12.4) |  |
| 2 | 36 | 3.9 (1.6-9.0) |  | 52 | 2.5 (1.4-7.2) |  |
| 3 | 5 | 4.9 (1.8-5.8) |  | 7 | 1.7 (1.0-5.5) |  |
| Inadequate sample | 0 |  |  | 1 |  |  |
| **TLR9 staining intensity, cytoplasm** |  |  |  |  |  |  |
| 0 | 0 | - | 0.473 | 4 | 6.6 (1.4-15.8) | 0.583 |
| 1 | 10 | 6.2 (1.4-20.1) |  | 54 | 3.5 (1.5-9.9) |  |
| 2 | 37 | 2.7 (1.5-7.1) |  | 34 | 2.6 (1.2-9.7) |  |
| 3 | 12 | 3.8 (1.1-8.5) |  | 11 | 4.1 (1.5-6.3) |  |
| Inadequate sample | 1 |  |  | 0 |  |  |
| **TLR9 staining intensity, membrane** |  |  |  |  |  |  |
| 0 | 16 | 1.7 (1.0-15.3) | 0.989 | 44 | 4.0 (1.6-13.8) | 0.371 |
| 1 | 11 | 3.9 (1.5-8.0) |  | 35 | 2.6 (1.3-6.8) |  |
| 2 | 24 | 4.6 (1.7-9.0) |  | 20 | 3.5 (1.4-10.7) |  |
| 3 | 8 | 2.5 (1.2-4.9) |  | 4 | 1.6 (1.1-7.0) |  |
| Inadequate sample | 1 |  |  | 0 |  |  |

Jonckheere-Terpstra test was used to determine the difference between median values. NAT=Neoadjuvant therapy, US=Upfront surgery, IQR=Interquartile range. Preoperative CRP was available in 60 NAT patients and 103 US patients. *Due to low patient number in this patient group, IQRs could not be calculated.
