# Supplementary material for: The expression and prognostic value of toll-like receptors (TLRs) in pancreatic cancer patients treated with neoadjuvant therapy
Source: PLoS One. 2022 May 10;17(5):e0267792. doi: 10.1371/journal.pone.0267792 (PMC9089880; doi:10.1371/journal.pone.0267792)
Supplement: S6 Table — Survival was calculated from surgery to disease progression or death due to pancreatic cancer. Survival was estimated with the Kaplan-Meier method (Log rank). NAT = Neoadjuvant therapy, US = Upfront surgery. *Due to low patient number with low TLR3 staining intensity, the 95% CI’s could not be calculated. (DOCX) [file pone.0267792.s006.docx]

**S6 Table. Disease-specific and disease-free survival in months according to TLR staining intensity for NAT and US patients separately.**

| **Staining** | **NAT (95% CI)**  **months** | ***p*-value**  **(log rank)** | **US (95% CI)**  **months** | ***p*-value**  **(log rank)** |
| --- | --- | --- | --- | --- |
| **TLR1** |  |  |  |  |
| *DSS* |  |  |  |  |
| 0-1 | **18 (5-32), n=13** | **0.029** | 21 (11-31), n=41 | 0.070 |
| 2-3 | **32 (29-35), n=57** |  | 30 (20-40), n=102 |  |
| *DFS* |  |  |  |  |
| 0-1 | 10 (7-12) | 0.140 | **9 (7-11)** | **0.047** |
| 2-3 | 16 (12-19) |  | **12 (7-17)** |  |
| **TLR2** |  |  |  |  |
| *DSS* |  |  |  |  |
| 0-1 | 43 (21-64), n=13 | 0.444 | 30 (1-60), n=21 | 0.802 |
| 2-3 | 30 (22-38), n=58 |  | 26 (19-33), n=122 |  |
| *DFS* |  |  |  |  |
| 0-1 | 11 (1-21) | 0.474 | 10 (3-16) | 0.781 |
| 2-3 | 14 (11-18) |  | 12 (8-15) |  |
| **TLR3** |  |  |  |  |
| *DSS* |  |  |  |  |
| 0-1 | 12 (-*), n=4 | 0.666 | 26 (16-37), n=45 | 0.668 |
| 2-3 | 30 (25-35), n=65 |  | 27 (17-37), n=98 |  |
| *DFS* |  |  |  |  |
| 0-1 | 7 (-)* | 0.440 | 9 (6-12) | 0.326 |
| 2-3 | 15 (11-18) |  | 13 (6-20) |  |
| **TLR4** |  |  |  |  |
| *DSS* |  |  |  |  |
| 0-1 | 30 (19-40), n=10 | 0.688 | 26 (10-42), n=22 | 0.885 |
| 2-3 | 30 (22-38), n=60 |  | 26 (18-35), n=121 |  |
| *DFS* |  |  |  |  |
| 0-1 | 9 (0-25) | 0.759 | 8 (2-14) | 0.884 |
| 2-3 | 14 (11-18) |  | 12 (9-15) |  |
| **TLR5** |  |  |  |  |
| *DSS* |  |  |  |  |
| 0-1 | 30 (22-37), n=35 | 0.789 | 23 (17-29), n=72 | 0.095 |
| 2-3 | 31 (22-40), n=36 |  | 31 (19-43), n=71 |  |
| *DFS* |  |  |  |  |
| 0-1 | 14 (7-21) | 0.429 | 10 (7-13) | 0.162 |
| 2-3 | 14 (10-19) |  | 14 (5-22) |  |
| **TLR7** |  |  |  |  |
| *DSS* |  |  |  |  |
| 0-1 | 31 (11-51), n=22 | 0.359 | **17 (11-23), n=60** | **0.002** |
| 2-3 | 30 (24-36), n=49 |  | **33 (18-48), n=83** |  |
| *DFS* |  |  |  |  |
| 0-1 | 14 (6-23) | 0.463 | **8 (6-10)** | **0.007** |
| 2-3 | 14 (10-18) |  | **16 (10-23)** |  |
| **TLR9 Cytoplasm** |  |  |  |  |
| *DSS* |  |  |  |  |
| 0-1 | 24 (9-39), n=11 | 0.567 | **25 (19-32), n=83** | **0.045** |
| 2-3 | 32 (29-35), n=59 |  | **38 (16-59), n=61** |  |
| *DFS* |  |  |  |  |
| 0-1 | 9 (5-14) | 0.757 | **9 (6-12)** | **0.044** |
| 2-3 | 16 (12-19) |  | **18 (13-23)** |  |
| **TLR9 Membranous** |  |  |  |  |
| *DSS* |  |  |  |  |
| 0-1 | 25 (19-32), n=33 | 0.988 | 26 (18-33), n=107 | 0.426 |
| 2-3 | 32 (30-33), n=37 |  | 33 (14-52), n=37 |  |
| *DFS* |  |  |  |  |
| 0-1 | 17 (8-25) | 0.380 | 10 (8-13) | 0.983 |
| 2-3 | 14 (11-17) |  | 16 (11-22) |  |

Survival was calculated from surgery to disease progression or death due to pancreatic cancer. Survival was estimated with the Kaplan-Meier method (Log rank). NAT=Neoadjuvant therapy, US=Upfront surgery, DSS=Disease-specific survival, DFS=disease-free survival. *Due to low patient number with low TLR3 staining intensity, the 95% CI’s could not be calculated.
