# Supplementary material for: The expression and prognostic value of toll-like receptors (TLRs) in pancreatic cancer patients treated with neoadjuvant therapy
Source: PLoS One. 2022 May 10;17(5):e0267792. doi: 10.1371/journal.pone.0267792 (PMC9089880; doi:10.1371/journal.pone.0267792)
Supplement: S7 Table — Univariate analysis for US and NAT patients separately. Univariate analysis calculated with the Cox proportional hazards method. End-point was disease-specific death. US = Upfront surgery, NAT = Neoadjuvant therapy. *Male vs female, **Stage IIB III vs IA IIA, ***TLR expression intensity high vs low. (DOCX) [file pone.0267792.s007.docx]

**S7 Table. Univariate analysis for US and NAT patients separately.**

|  | **HR (95% CI)** | ***p*-value** |
| --- | --- | --- |
| **US** |  |  |
| Age | 1.01 (0.99-1.04) | 0.264 |
| Sex* | 1.14 (0.79-1.65) | 0.481 |
| Stage** | 2.05 (1.32-3.20) | **0.001** |
| Adjuvant therapy*** | 0.69 (0.47-1.01) | 0.055 |
| Perineural invasion | 1.19 (0.76-1.87) | 0.444 |
| Perivascular invasion | 2.46 (1.68-3.60) | **<0.001** |
| Radical resection | 0.73 (0.47-1.13) | 0.159 |
| Vascular resection | 1.29 (0.87-1.90) | 0.203 |
| Tumor grade, 1 | - | **0.001** |
| 2 | 1.28 (0.76-2.14) | 0.349 |
| 3 | 2.99 (1.59-5.60) | **0.001** |
| TLR**** |  |  |
| 1 | 0.69 (0.46-1.03) | 0.072 |
| 2 | 1.07 (0.63-1.82) | 0.802 |
| 3 | 0.92 (0.62-1.36) | 0.668 |
| 4 | 0.95 (0.58-1.58) | 0.855 |
| 5 | 0.73 (0.50-1.06) | 0.096 |
| 7 | 0.57 (0.39-0.82) | **0.003** |
| 9 cytoplasm | 0.68 (0.47-0.99) | **0.047** |
| 9 membrane | 0.84 (0.55-1.29) | 0.426 |
| **NAT** |  |  |
| Age | 1.03 (1.00-1.06) | 0.076 |
| Sex* | 1.27 (0.73-2.20) | 0.399 |
| Stage** | 1.19 (0.69-2.03) | 0.538 |
| Adjuvant therapy*** | 0.41 (0.24-0.71) | **0.001** |
| Perineural invasion | 1.65 (0.91-2.98) | 0.097 |
| Perivascular invasion | 1.34 (0.71-2.52) | 0.364 |
| Radical resection | 0.62 (0.31-1.24) | 0.173 |
| Vascular resection | 0.93 (0.54-1.60) | 0.788 |
| Tumor grade, 1 | - | 0.885 |
| 2 | 1.20 (0.59-2.42) | 0.621 |
| 3 | 1.16 (0.48-2.81) | 0.738 |
| TLR**** |  |  |
| 1 | 0.49 (0.26-0.94) | **0.032** |
| 2 | 1.32 (0.64-2.72) | 0.446 |
| 3 | 1.36 (0.33-5.62) | 0.667 |
| 4 | 1.18 (0.53-2.62) | 0.689 |
| 5 | 0.93 (0.54-1.59) | 0.789 |
| 7 | 1.32 (0.73-2.38) | 0.361 |
| 9 cytoplasm | 0.80 (0.38-1.72) | 0.568 |
| 9 membrane | 1.00 (0.58-1.72) | 0.988 |

Univariate analysis calculated with the Cox proportional hazards method. End-point was disease-specific death. US=Upfront surgery, NAT=Neoadjuvant therapy, HR=Hazards ratio.

*Male vs female

**Stage IIB III vs IA IIA

***Adjuvant therapy after surgery, yes vs no

****TLR expression intensity high vs low.
