# Supplementary material for: The expression and prognostic value of toll-like receptors (TLRs) in pancreatic cancer patients treated with neoadjuvant therapy
Source: PLoS One. 2022 May 10;17(5):e0267792. doi: 10.1371/journal.pone.0267792 (PMC9089880; doi:10.1371/journal.pone.0267792)
Supplement: S8 Table — NAT and US patients were analyzed separately. Multivariate model including TLR expression intensity (for each TLR separately), age, sex, stage, adjuvant therapy and perivascular invasion. 1p<0.001, 2p<0.01, 3p<0.05. *Sex male vs female, **Stage IIB-III vs IA-IIA, ***TLR high expression intensity vs low expression intensity. (DOCX) [file pone.0267792.s008.docx]

**S8 Table. Multivariate analysis for each TLR separately. NAT and US patients were analyzed separately.**

|  | **TLR1 HR (95% CI)** | **TLR2 HR (95% CI)** | **TLR3 HR (95% CI)** | **TLR4 HR (95% CI)** | **TLR5 HR (95% CI)** | **TLR7 HR (95% CI)** | **TLR9 cytoplasm HR (95% CI)** | **TLR9 membrane HR (95% CI)** |
| --- | --- | --- | --- | --- | --- | --- | --- | --- |
| **US** |  |  |  |  |  |  |  |  |
| Age | 1.01 (0.98-1.04) | 1.01 (0.99-1.04) | 1.01 (0.98-1.04) | 1.01 (0.98-1.04) | 1.01 (0.98-1.04) | 1.01 (0.99-1.04) | 1.01 (0.98-1.04) | 1.01 (0.99-1.04) |
| Sex* | 1.03 (0.65-1.43) | 0.98 (0.67-1.46) | 0.98 (0.66-1.45) | 0.99 (0.67-1.46) | 1.02 (0.69-1.51) | 0.98 (0.66-1.45) | 0.96 (0.65-1.43) | 1.01 (0.68-1.50) |
| Stage** | **1.98 (1.19-3.30) ^2^** | **2.00 (1.20-3.35) ^2^** | **2.00 (1.19-3.34) ^2^** | **1.97 (1.18-3.30) ^3^** | **2.12 (1.27-3.52) ^2^** | **1.74 (1.03-2.93) ^3^** | **2.01 (1.20-3.35) ^2^** | **2.00 (1.20-3.36) ^2^** |
| Adjuvant  Therapy*** | **0.49 (0.33-0.74) ^1^** | **0.49 (0.33-0.74) ^2^** | **0.49 (0.33-0.74) ^2^** | **0.49 (0.23-0.74) ^2^** | **0.46 (0.31-0.69) ^1^** | **0.48 (0.32-0.73) ^2^** | **0.49 (0.33-0.74) ^2^** | **0.50 (0.33-0.75) ^2^** |
| Perivascular invasion | **2.17 (1.43-3.29) ^1^** | **2.16 (1.41-3.30) ^1^** | **2.15 (1.41-3.27) ^1^** | **2.18 (1.43-3.34) ^1^** | **2.19 (1.45-3.31) ^1^** | **2.27 (1.50-3.49) ^1^** | **2.07 (1.37-3.15) ^2^** | **2.23 (1.46-3.42) ^1^** |
| TLR **** | 0.66 (0.44-1.00) **^4^** | 0.91 (0.53-1.58) | 0.87 (0.59-1.30) | 0.85 (0.51-1.43) | **0.63 (0.43-0.92) ^3^** | **0.59 (0.40-0.87) ^2^** | 0.73 (0.50-1.07) | 0.72 (0.47-1.12) |
| **NAT** |  |  |  |  |  |  |  |  |
| Age | 1.02 (0.98-1.05) | 1.02 (0.99-1.05) | 1.02 (0.99-1.06) | 1.02 (0.99-1.05) | 1.02 (0.99-1.05) | 1.02 (0.99-1.05) | 1.02 (0.99-1.05) | 1.02 (0.99-1.05) |
| Sex* | 1.32 (0.73-2.38) | 1.34 (0.76-2.54) | 1.40 (0.77-2.57) | 1.33 (0.74-2.39) | 1.03 (0.74-2.31) | 1.35 (0.75-2.41) | 1.39 (0.77-2.51) | 1.40 (0.77-2.53) |
| Stage** | 1.26 (0.71-2.24) | 1.18 (0.66-2.12) | 1.10 (0.62-1.99) | 1.19 (0.62-1.98) | 1.22 (0.69-2.17) | 1.22 (0.68-2.20) | 1.15 (0.64-2.07) | 1.17 (0.65-2.10) |
| Adjuvant  Therapy*** | **0.40 (0.22-0.73) ^2^** | **0.43 (0.24-0.75) ^2^** | **0.39 (0.22-0.71) ^2^** | **0.36 (0.19-0.66) ^2^** | **0.43 (0.24-0.76) ^2^** | **0.41 (0.23-0.74) ^2^** | **0.39 (0.22-0.72) ^2^** | **0.40 (0.22-0.72) ^2^** |
| Perivascular  invasion | 0.90 (0.45-1.81) | 0.87 (0.42-1.80) | 0.87 (0.42-1.80) | 0.81 (0.40-1.66) | 0.99 (0.47-2.07) | 0.87 (0.42-1.80) | 0.87 (0.42-1.80) | 0.90 (0.43-1.85) |
| TLR**** | **0.48 (0.25-0.95)^3^** | 1.32 (0.60-2.91) | 1.51 (0.34-6.79) | 1.56 (0.64-3.80) | 0.88 (0.49-1.59) | 1.33 (0.73-2.44) | 1.03 (0.45-2.34) | 0.93 (0.50-1.71) |

Multivariate model including TLR expression intensity (for each TLR separately), age, sex, stage, adjuvant therapy and perivascular invasion. US=Upfront surgery, NAT=Neoadjuvant therapy, HR=Hazards ratio. ^1^p<0.001, ^2^p<0.01, ^3^p<0.05, ^4^p=0.050

*Sex male vs female

**Stage IIB-III vs IA-IIA

***Adjuvant therapy after surgery vs no adjuvant therapy after surgery

****TLR high expression intensity vs low expression intensity
