# Supplementary material for: The expression and prognostic value of toll-like receptors (TLRs) in pancreatic cancer patients treated with neoadjuvant therapy
Source: PLoS One. 2022 May 10;17(5):e0267792. doi: 10.1371/journal.pone.0267792 (PMC9089880; doi:10.1371/journal.pone.0267792)
Supplement: S9 Table — NAT and US patients analyzed separately. (DOCX) [file pone.0267792.s009.docx]

**S9 Table. Additional multivariate model with TLR7 and TLR9 combined.**

| **US** | **HR (95% CI)** | **p-value** |
| --- | --- | --- |
| **Age** | 1.01 (0.98-1.04) | 0,454 |
| **Sex*** | 0.99 (0.67-1.47) | 0,957 |
| **Stage**** | 1.80 (1.08-3.01) | 0,025 |
| **Adjuvant therapy***** | 0.50 (0.33-0.75) | 0,001 |
| **Perivascular invasion** | 2.23 (1.47-3.40) | <0,001 |
| **TLR7 and TLR9 low** | ref | 0.019 |
| TLR7 or TLR9 high | 0.66 (0.42-1.03) | 0.066 |
| TLR7 and TLR9 high | 0.50 (0.31-0.82) | 0.006 |
| **NAT** |  |  |
| **Age** | 1.02 (0.99-1.06) | 0.230 |
| **Sex*** | 1.32 (0.72-2.42) | 0.362 |
| **Stage**** | 1.16 (0.64-2.10) | 0.632 |
| **Adjuvant therapy***** | 0.40 (0.22-0.73) | 0.003 |
| **Perivascular invasion** | 0.805 (0.38-1.72) | 0.575 |
| **TLR7 and TLR9 low** | ref | 0.350 |
| TLR7 or TLR9 high | 0.53 (0.16-1.70) | 0.282 |
| TLR7 and TLR9 high | 0.80 (0.26-2.47) | 0.696 |

HR=Hazards ratio, CI=Confidence interval, US=upfront surgery, NAT=neoadjuvant therapy.

*Sex male vs female

**Stage IIB-III vs IA-IIA

***Adjuvant therapy after surgery vs no adjuvant therapy after surgery
